# Going underground: short- and long-term movements may reveal the fossorial spatial ecology of an amphisbaenian

**DOI:** 10.1186/s40462-021-00253-x

**Published:** 2021-03-23

**Authors:** José Martín, Jesús Ortega, Roberto García-Roa, Octavio Jiménez-Robles, Gonzalo Rodríguez-Ruiz, Pablo Recio, José Javier Cuervo

**Affiliations:** 1 Departamento de Ecología Evolutiva, Museo Nacional de Ciencias Naturales, CSIC, Madrid, Spain; 2 Institute of Evolutionary Biology, CSIC-Universitat Pompeu Fabra, Barcelona, Spain; 3 Ethology Lab, Cavanilles Institute of Biodiversity and Evolutionary Biology, University of Valencia, Valencia, Spain; 4 Department of Ecology and Evolution, Research School of Biology, Australian National University, Canberra, Australia

**Keywords:** Amphisbaenians, Fossorial reptiles, Movement patterns, PIT tag telemetry, Space use, *Trogonophis wiegmanni*

## Abstract

**Background:**

The movement and spatial ecology of an animal depends on its morphological and functional adaptations to its environment. In fossorial animals, adaptations to the underground life help to face peculiar ecological challenges, very different from those of epigeal species, but may constrain their movement ability.

**Methods:**

We made a long-term capture-recapture study of the strictly fossorial amphisbaenian reptile *Trogonophis wiegmanni* to analyze its long-term movement patterns. We also used passive integrated transponder (PIT) telemetry to detect and follow undisturbed individuals underground, obtaining data of their short-term movement patterns.

**Results:**

Amphisbaenians showed a high site fidelity, moving short distances and over small areas, and spending some days without any noticeable movement, even under favorable conditions. We also found differences in movements between sexes and age classes.

**Conclusions:**

This movement and spatial strategy can be related to the energetic constrains of underground burrowing, or to the low metabolic requirements of fossorial reptiles, as distances and areas covered were much smaller than for epigeal reptiles of similar size. Individual differences probably reflect differential reproductive and social requirements of males and females, and that younger individuals might show more floating behavior until they can settle in a territory. This study is a rare example describing the movement ecology of a fossorial species and may contribute to the general understanding of the factors that affect space use and movement decisions in animals.

## Introduction

Animals do not move and use all the available space at random but following specific ecological patterns that determine the structure and dynamics of each species and the entire community [1, 2]. The observed variation in movement and space use strategies may be explained by physiological constraints, such as energetic, foraging and reproductive requirements, or the ability for moving in determined habitats [3–9]. Also, there may be ecological constraints, such as the density of conspecifics/heterospecifics, or other environmental factors (e.g. availability of optimal habitats, etc). These factors determine that some species or classes of individuals are more territorial or sedentary, showing high site fidelity to a small area, while others behave as floaters or nomadic, and move widely around large areas [10–12]. Furthermore, within the same species, these variations are often associated with sexual or ontogenetic differences [10–12]; for example, males move more often and have larger territories to search and have access to more females, whereas females’ home ranges are generally smaller because they primarily depend on food resources [5]. Also juveniles may be more nomadic, dispersing over large areas before they settle as adults in a given territory [9, 13]. For these reasons, it is important to study a broad picture, including microhabitat selection, movement patterns and home ranges, to understand what drives the animal movements and space use strategies [14, 15].

Moreover, to understand the general patterns in animal movement and spatial ecology, we should examine a large number of species with different adaptations and types of life styles and not only the most conspicuous or easy to study. However, among vertebrates, because fossorial reptiles spend all or most of their lives underground, their biology and ecology are much less known than those of their epigeal relatives. This is an oversight because fossorial reptiles are nearly 30% of the reptile species of the world (i.e., more than 2000 species, including many skinks, legless lizards, blind snakes and amphisbaenians) [16, 17]. This lack of information may be explained by the apparent low population densities of fossorial reptile species and the difficulty of finding, sampling and observing their behavior [16, 18]. Remarkably, the study of the movement ecology of fossorial animals is also essential if we aim to gain a better grasp on the ecological challenges they withstand, which, as a consequence of their characteristic adaptations to the underground life, might significantly differ from those of epigeal species [19, 20].

One of the most prominent, but also inconspicuous and understudied, groups of fossorial reptiles are the amphisbaenians [20, 21]. These reptiles have very specialized morphological and functional adaptations to burrow and feed successfully underground, such as reduced vision, narrow heads or loss of limbs [20–23]. These adaptations, however, constrain many aspects of their ecology [24–27], bringing along challenges for moving underground over large distances and having large home ranges. This might be expected due to the energetic costs of burrowing [28] and the difficulty of underground orientation and navigation. Unfortunately, our understanding about amphisbaenians movement ecology is still very meager.

Here, we studied the movement and spatial ecology of the fossorial Checkerboard worm lizard, *Trogonophis wiegmanni*, an amphisbaenian found in the NW African Mediterranean [29]. Similarly to other amphisbaenians, it spends all of its life underground, which likely explains the lack of information available for its movement ecology. However, there is increasing knowledge on its habitat selection patterns [30, 31], thermal biology [32, 33], feeding ecology [23, 34–36], reproduction [34], population and social biology [37–40] and conservation problems [41–43].

We used data from a long-term capture-recapture study (2015–2020) of island populations of *T. wiegmanni* amphisbaenians that provided an insight into the long-term movement patterns of individuals recaptured under rocks. We also used passive integrated transponder (PIT) telemetry (i.e., detecting at a distance the radiofrequency signal of pit-tag-marked buried individuals) [44–46] to detect and follow undisturbed individuals underground, obtaining data of their short-term movement patterns. We hypothesized that the fossorial environment may constraint movement rates, showing amphisbaenians a high site fidelity. We tested this hypothesis between sexes and age classes. We expected that differential reproductive and social requirements of males and females might result in intersexual differences in their movement and spatial patterns, with males moving more frequently and over longer distances than females. We also expected that younger subadult individuals might show more floating behavior until they can settle in a territory.

## Materials and methods

### Study area

We carried out this study at the archipelago of Chafarinas Islands (Spain) (35°11′ N, 02° 25′ W), located in the southwestern Mediterranean Sea, close to the northern Moroccan coast (2.5 nautical miles offshore Ras el Ma). Vegetation is dominated by woody bushes (*Salsola*, *Suaeda*, *Lycium* and *Atriplex*), which are adapted to soil salinity and drought resulting from an arid and warm Mediterranean climate [47]. Soils are shallow, poorly developed and immature with a thin A horizon, rich in organic matter, underlain by the original volcanic rock [48]. The amphisbaenian *T. wiegmanni* is very abundant at this archipelago [49, 50].

### Sampling procedures

We visited the Chafarinas Islands in spring (March–April) and early autumn (September–October) from 2015 to 2020 during ten field campaigns of 2 weeks duration each. We delimited three study plots in different islands: Isabel (area of the plot = 0.14 Ha), Northern part of Rey (0.40 Ha) and Southern part of Rey (0.58 Ha). The plots were delimited following geographical feature limitations and comprised areas with homogeneous habitat conditions, which were optimal for amphisbaenians [31], allowing them to occupy all the surface of the plots, We walked systematically and intensively the plots during the morning and afternoon of different days. Each plot was surveyed completely 2–3 times in each campaign, with larger plots requiring more time to be completed. We searched for amphisbaenians by carefully lifting almost all rocks and stones found inside the plots. Amphisbaenians were common and easy to find under rocks [31, 49], which were abundant enough (> 40% of rock surface cover) to allow a large effective survey area. Individuals were captured by hand and, on the spot, measured (snout-to-vent length = SVL, tail length and body weight) and sexed by examining the presence of hemipenes in the cloacae [38, 39]. As it usually occurs in reptiles, a previous study [38] showed that different age classes of this amphisbaenian are characterized by different body sizes, with younger individuals being clearly smaller than older individuals. Thus, we used body size (SVL) as a proxy of age in analyses.

Immediately after taking measurements, we marked amphisbaenians at first capture by implanting PIT tags (8.4 mm × 1.4 mm; Biomark MiniHPT8; Biomark, Inc., Boise, Idaho, USA) subcutaneously in the upper right side of the body [51, 52]. Due to the size of the tags, only amphisbaenians with a SVL longer than 90 mm (i.e. second year subadults; see [38]) could be marked (for details and validation of the procedure in this amphisbaenian see [52]). When captured, we used a hand-held portable reader (Biomark 601 Reader) to test if the individual was already marked and, in that case, read the individual unique code of the tag, or marked it if unmarked. The location of each individual was determined with a GPS (GPSmap 62st; Garmin Ltd., Olathe, Kansas, USA) that included a Quadrifilar Helix antenna. Each position was recorded for 45 s to increase accuracy (± 3 m) and the GPS was previously calibrated respect to reference points in each session to decrease measurement error, given the accuracy limitations of GPS systems. We released amphisbaenians at their exact point of capture in less than 5 min after finding them.

### PIT telemetry

In March 2020, we also surveyed during several days the entire surface of the study plots using a HPR Plus Reader equipped with a BP Plus Lite Portable Antenna (Biomark, Inc., Boise, Idaho, USA). This reader allowed telemetry detection of PIT tags of marked amphisbaenians while they remain buried in any place under the ground surface, without the need to excavate to bring them to the surface or find them under a lifted rock (for the use of this method in other fossorial animals see [44–46]. Thus, we avoided any possible disturbance to individuals and any possible bias related to surveys restricted to rocks. Preliminary trials showed that buried 8.4 mm PIT tags could be detected up to about 20 cm deep (unpublished data; see also [46]). In each session, the study plot was surveyed in a linear search pattern beginning at the southern end and moving to the northern side of the plot. While searching, the antenna was slowly swung from side to side giving sufficient overlap to cover the ground completely. All plots were searched as thoroughly as possible, including all open ground areas, rocks and by inserting the antenna into the bushes basis. After a PIT tag marked amphisbaenian was initially detected and located, the individual’s position was determined with the GPS and the exact location marked with a surveying flag labeled with the tag number.

We further used this procedure to study daily short-term underground movements of amphisbaenians only in the plot located in Isabel Island. As tracked individuals remained underground and were not handled or disturbed by us during these surveys, we consider that they moved freely, independently of our short-term repeated surveys. We visited the plot for seven successive days from 7th to 14th March 2020. After the initial detection, during each subsequent visit, we tracked and tried to relocate all previously detected individuals. If an amphisbaenian was not found at the previously known location marked with a flag, we searched for it with the antenna, starting at the last point detected and continuing in a circular pattern. If it was not detected in a circle of 3 m radius around the last point, we considered it was beyond the depth range of the device or had moved far away, and the individual was noted as missed. If the individual had moved and was detected, the shortest lineal distance from the previous to the current position was measured with a metric tape to the nearest cm, and the direction (angle) of this line with respect to the North cardinal point measured with a magnetic compass to the nearest 10°. The flag was repositioned to the new location and the time spent between successive relocations was also noted. While we were searching for these individuals in areas around the flags, we often detected other previously located but missed individuals or new marked individuals that were incorporated to the study.

At the end of the surveys, we gently excavated at the final location of each individual to try to recapture it. In this way, we first ensured that individuals that had not moved from the initial location were alive. This is because the PIT tag remained underground after an individual had died and could actually be detected using the reader. If we found a PIT tag, alone or inside the remains of a dead amphisbaenian, we discarded its data in this study. Recaptured live individuals were measured and returned to their locations immediately. For a few marked individuals that we followed but could not recapture at the end, we estimated their predicted current body size using measurements from their last previous recaptures, the time spent since these recaptures until the current detection, and the mean growth rate for that size/age class obtained from long-term multiple recapture data of many individuals in that population (unpublished data). Similar calculations comparing predicted values with actual measurements of some of the recaptured individuals showed a high predictive value (Spearman’s correlation, *r*_*s*_ = 0.76, *n* = 53, *P* < 0.0001).

### Analysis of movement data

To study the movements of amphisbaenians, we used three approaches. First, we studied long-term movements of an individual by calculating the shortest lineal distance (to the nearest 1 m) between the first capture and the successive recaptures under rocks made in different field campaigns. These distances did not represent the total distance moved by an individual in a given long-term period, as individuals may do multiple movements and follow non-lineal paths. However, we considered this distance as a measure of long-term fidelity to the initial capture point. If individuals moved randomly and over larger areas, then the probability of being recaptured close to the initial point would be low. On the contrary, if individuals showed high site fidelity and moved over small areas, or returned to the same point after moving, then, the probability of being recaptured close to the initial point would be high.

We used a second approach because rocks could attract animals for thermoregulation and foraging [33, 53] and, then, recaptures restricted to rocks might bias the actual movement patterns to other places underground. Thus, we calculated the long-term shortest lineal distance (to the nearest 1 m) moved by each individual between its first capture locations under rocks (in previous campaigns) and the location of the point where it was first detected with the reader in march 2020 in any place underground all over the surface of the study plots.

For analyzing these long-term movements, we used three separated Lineal Mixed Models (LMMs) using as dependent variables with a normal distribution either i) the distance from capture to first recapture under rocks in a different field campaign, ii) the mean distance between successive recaptures of the same individual under rocks in different field campaigns, or iii) the distance from first capture to detection in 2020 when being underground in any place. We tested for differences between sexes (fixed factor) or with body size (SVL) (continuous variable) and considered the interaction between sex and body size. We also included in the models the study plot as a random factor, and the time between recaptures as a continuous covariate to control for a possible effect of time on distance moved. To characterize body size, we used SVL of individuals at first capture, but repeating the LMMs with size at recapture, or the mean size between values at capture and first recapture, yielded qualitatively identical results in all cases (results not shown).

Finally, within the 2020 spring campaign, we calculated short-term movements (to the nearest 1 cm) of individuals relocated in any place of only one of the plots (Isabel) at least twice while they remained underground and undisturbed. For these short-term movements, given the skewed non-normal distribution of data, we used three different Generalized Linear Models (GLZs), with a Poisson distribution and a log link function, with the dependent variables being either i) the mean distance moved per day, or ii) the percentage of days with movements, or iii) the mean distance moved considering only the days in which a meaningful movement occurred (i.e., excluding days without movements). We included in the models the sex as explanatory fixed factor, body size (SVL) as a continuous covariate, and the interaction between sex and body size. When this interaction was significant, we made further separated GLZ models for males and females to explore the meaning and direction of such interaction.

During the March 2020 campaign, we used the minimum convex polygon (MCP) method [54] to assess the short-term area covered by individual amphisbaenians that were followed every day while they moved undisturbed underground. This area was not considered to be equivalent to an entire home range, given the low number of independent locations used to estimate it and the limitations of this method [55], However, this area was considered only as an indicator of the surface that an amphisbaenian could cover in a few days. To estimate this area with independent observations, we only used individuals with at least three different locations in three different days (only the first one of each day) (mean ± SE = 3.2 ± 0.1 points; range = 3–5; *n* = 23 individuals). The size of the area was not significantly related to the number of points used to estimate it (Spearman’s correlation, *r*_*s*_ = 0.22, *n* = 23, *P* = 0.30).

Because of the skewed non-normal distribution of area data, we used a Generalized Linear Model (GLZ), with a Poisson distribution and a log link function, with the the short-term areas covered as the dependent variables, using the sex as explanatory fixed factor, body size (SVL) as a continuous variable, and its two-way interaction, and the, the number of points used to estimate the area as a continuous covariate.

## Results

### Long-term movements based on recaptures under rocks

The distance between the location where an amphisbaenian was first captured and the location of its first recapture under rocks in a different field campaign (time interval, mean ± SE = 488 ± 27 days) was on average (± SE) of 4.4 ± 0.2 m (range = 0–16 m, *n* = 166 individuals). Nevertheless, considering the accuracy limits of the GPS, the actual mean value of the distance between recpatures might oscillate between 0 and 10 m, and the range of values might increase until 22 m in one individual, for the “worst” possible mistake in location measurements. This distance did not vary significantly between sexes or with body size in any of the study plots (i.e. among islands), and it was independent of the time interval between the first capture and the first recapture (Table 1). Similarly, the mean distance among all successive recaptures under rocks of an amphisbaenian in different campaigns (only the first recapture in every campaign) was of 4.3 ± 0.2 m, and it did not vary significantly between sexes, sizes or plots (Table 1).

**Table 1:** Long-term movements of amphisbaenians based on recaptures under rocks (*n* = 167) or on detections with the reader underground in any place (*n* = 137). Results of LMMs testing the effects of sex (fixed factor), body size (continuous variable) and study plot (random factor) on the distance between locations, including time between recaptures as a covariate

	Distance from initial capture to1st recapture under rocks			Mean distance between recaptures under rocks			Distance from initial capture to detection underground in any place		
	*F*	df	*P*	*F*	df	*P*	*F*	df	*P*
Sex	0.81	1	0.37	0.06	1	0.81	0.69	1	0.41
Body size	0.16	1	0.67	0.28	1	0.60	0.12	1	0.73
Study plot	2.20	2	0.11	2.71	2	0.07	2.22	2	0.11
Time interval	0.32	1	0.57	0.02	1	0.83	2.61	1	0.11
Sex x Size	1.04	1	0.31	0.14	1	0.70	0.82	1	0.37
Error		161			161			130	

### Long-term movements based on detection of individuals underground

The distance between the location where an amphisbaenian was first captured under rocks and the location where it was detected with the reader underground in any place of the plot (time interval, mean ± SE = 832 ± 43 days) was on average (± SE) of 4.4 ± 0.2 m (range = 0–10.2 m, *n* = 137 individuals). Similarly to recaptures under rocks and considering the GPS limitations, the actual mean distance might oscillate between 0 and 10 m. This distance did not vary significantly between sexes or with body size in any of the study plots, and it was independent of the time interval between the first capture and the detection (Table 1).

### Short-term movements

**Fig. 1:**
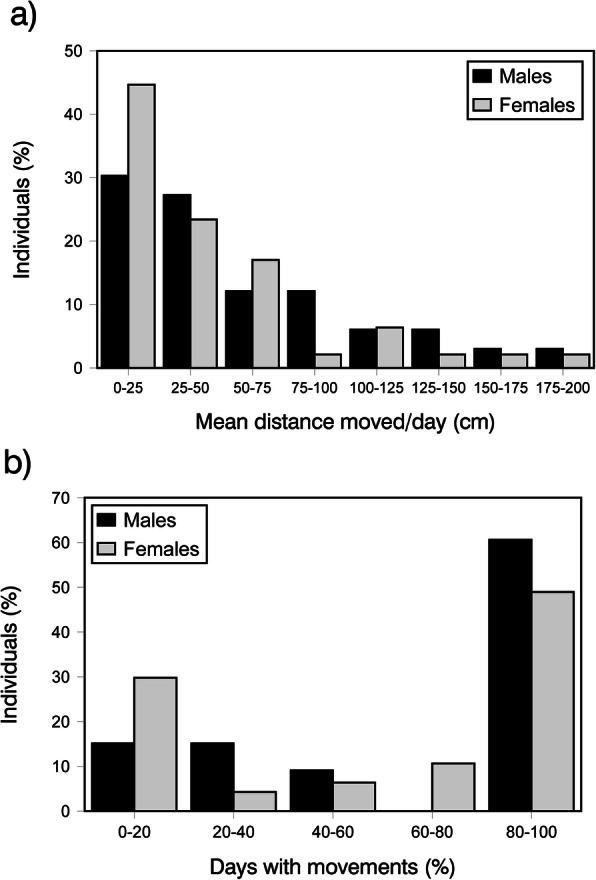
Short-term underground movements of undisturbed amphisbaenians based on daily detections with the reader in any place during seven consecutive days. Frequency distribution of individuals (% within each sex; *n* = 47 females and 33 males) in each category of **a** mean distance moved per day (cm) and **b** percentage of days in which some meaningful movements occurred

The distance moved underground in successive days by undisturbed amphisbaenians that were followed with the reader was on average (± SE) of 46 ± 5 cm/day (range = 0–200 cm/day, *n* = 80 individuals) (Fig. 1a). Distances moved by males (54 ± 9 cm/day; *n* = 33) were significantly longer than those moved by females (40 ± 7 cm/day, *n* = 47; GLZ: χ^2^ = 75.14, *P <* 0.0001), and although there was not an overall significant effect of body size (χ^2^ = 0.45, *P =* 0.50), its interaction with sex was significant (sex x size, χ^2^ = 323.84, *P <* 0.0001). To explore the meaning of this interaction, we made further separated GLZs models for males and females, which suggested that smaller (younger) males moved for longer distances than larger (older) ones (GLZ: χ^2^ = 144.90, *P <* 0.0001, *Estimate* = − 0.014 ± 0.001), while the opposite relationship was observed in females (GLZ: χ^2^ = 179.39, *P <* 0.0003, *Estimate* = 0.019 ± 0.001).

However, in many cases amphisbaenians did not seem to change their location from 1 day to the other in a meaningful way, even if, as it occurs during all our surveys, thermal conditions were favorable for activity and other individuals were moving. Thus, average moving distance per day was positively correlated with number of days in which movements occurred (*r*_*s*_ = 0.78, *n* = 80, *P* < 0.0001). The percentage of days on which we detected movements were on average (± SE) of 65 ± 5% (range = 0–100%, *n* = 80 individuals) (Fig. 1b). Males moved a significantly higher number of days than females (71 ± 7% vs. 61 ± 6%, GLZ: χ^2^ = 29.51, *P <* 0.0001), body size was not significant (χ^2^ = 0.64, *P =* 0.42), but the interaction was significant (sex x size, χ^2^ = 77.21, *P <* 0.0001). To explore the meaning of this interaction, we made further separated GLZs for males and females, which suggested that smaller males moved during more days than larger ones (GLZ: χ^2^ = 5.57, *P =* 0.018, *Estimate* = − 0.001 ± 0.001), while the opposite relationship occur in females (GLZ: χ^2^ = 36.18, *P <* 0.0001, *Estimate* = 0.007 ± 0.001).

When we considered only the distances moved greater than zero (i.e., excluding days without apparent movements and 19 individuals that did not seem to move in any day), the mean distance was on average (± SE) of 80 ± 8 cm/day (range = 15–332 cm/day, *n* = 61 individuals). This distance was significantly longer (GLZ: χ^2^ = 76.54, *P <* 0.0001) in males (91 ± 13 cm/day, *n* = 27) than in females (71 ± 9 cm/day, *n* = 34), and there was an overall significant effect of body size (χ^2^ = 14.88, *P =* 0.0001), but the interaction was significant (sex x size, χ^2^ = 74.87, *P <* 0.0001). Further separated GLZs for males and females suggested again that, considering only the days that actually moved, the smallest males moved significantly longer distances than larger ones (GLZ: χ^2^ = 42.35, *P <* 0.0001, *Estimate* = − 0.008 ± 0.001), while the converse occurred in females (GLZ: χ^2^ = 80.79, *P <* 0.0001, *Estimate* = 0.012 ± 0.001).

**Fig. 2:**
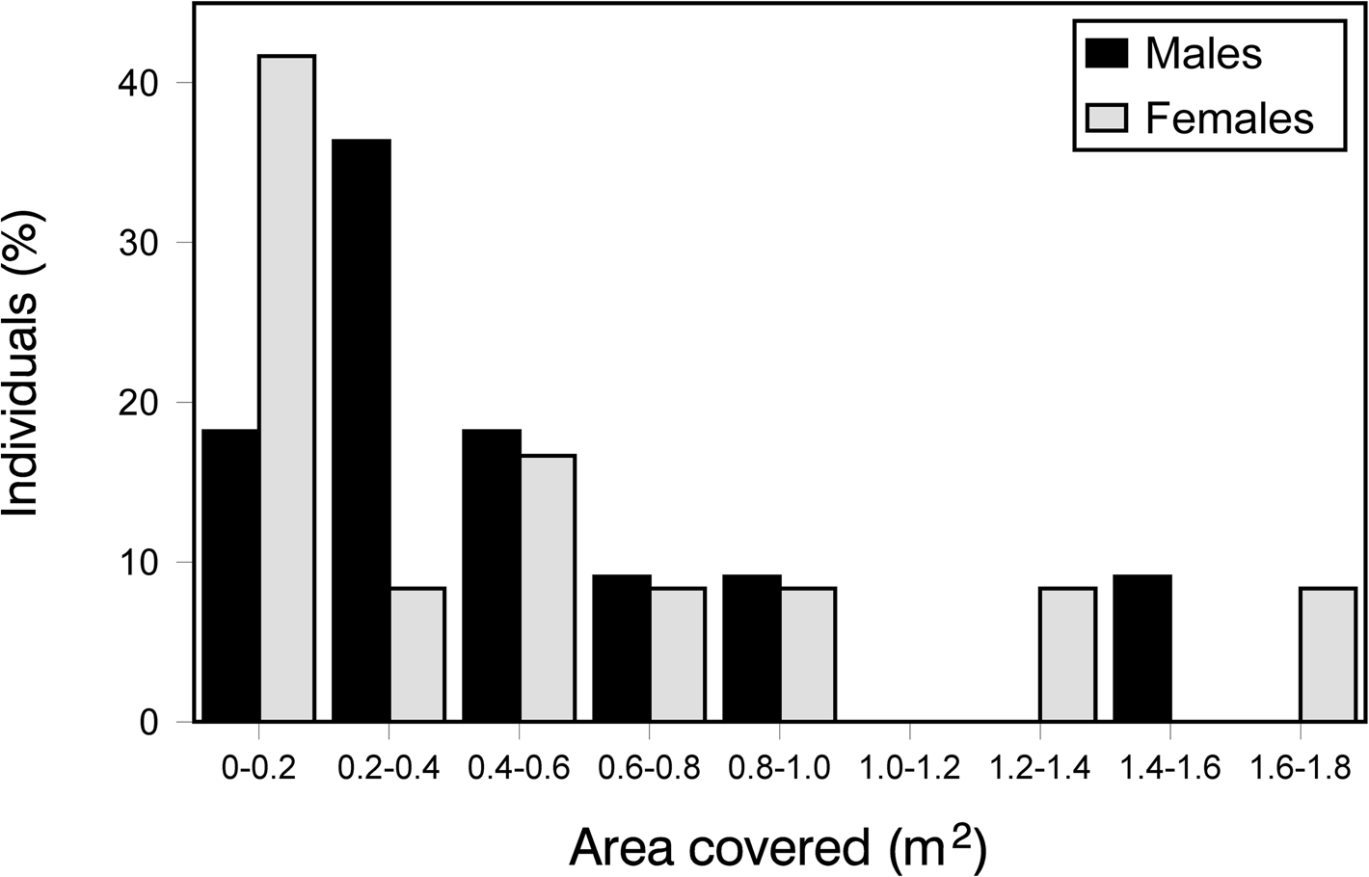
Areas covered by amphisbaenians in the short-term when moving underground in successive days. Frequency distribution of individuals (% within each sex; *n* = 11 females and 12 males) in each category of area (m^2^) estimated from detections made in different successive days (one point per day)

The total area covered by amphisbaenians during these short-term successive movements was on average (± SE) of 0.50 ± 0.10 m^2^ (range = 0.02–1.68 m^2^, *n* = 23) (Fig. 2). This area did not vary significantly with sex (GLZ: χ^2^ = 0.08, *P =* 0.77), body size (χ^2^ = 0.95, *P =* 0.33), the interaction between sex and size was not significant (χ ^2^ = 0.41, *P =* 0.52), and the size of the area was independent of the number of points used to estimate it (χ^2^ = 0.05, *P =* 0.83).

## Discussion

Our study provides, for the first time, some insights of the underground movement and spatial ecology of an amphisbaenian species. Moreover, these results contribute to further our knowledge of the movement ecology of fossorial reptiles, which has been largely understudied. Long-term recaptures indicated that *T. wiegmanni* amphisbaenians showed high site fidelity with limited displacements, considering the distance that they move away from initial capture points Short-term movement patterns also indicated short distances moved (but not necessarily slow movement rates) and small areas covered. Short-term data further showed the existence of intersexual and ontogenetic differences probably reflecting different requirements and strategies of movement and space use.

The long-term recapture observations indicated that *T. wiegmanni* amphisbaenians did not move very far from the initial capture point, even after many days. Also, short-term daily observations showed short movements over small areas in successive days. Thus, although we could not have estimations of actual home range sizes, our data suggest that these home ranges must be very small (just a few m^2^) in comparison with epigeal reptiles of similar body size. For example, average home range size is 0.04 ha in *Podarcis* wall lizards [56], 0.09 ha in *Zootoca vivipara* lizards [57], 0.07 ha in smooth snakes, *Coronella austriaca* [58], 0.38 ha in the slow worm *Anguis fragilis* [59]. In contrast with epigeal species, the fossorial lizard *Anniella pulchra*, that inhabits sand dunes where burrowing is easy, had relatively small home ranges (around 0.007 ha for a 95% Kernel and 0.0016 for a 50% Kernel), although with a high interindividual variability (between less than 0.001 and 0.02 ha) [44]. Similarly, the semi-fossorial worm snake, *Carphophis amoenus*, also has limited movement rates, although not so small home ranges (0.025 ha) [60, 61].

The most plausible explanation for these differences between epigeal and fossorial reptiles may be that underground burrowing movements are energetically costly for fossorial reptiles [19, 22, 28], but see [62], as movements are also costly for subterranean rodents [63, 64]. Probably due to these locomotory constraints, amphisbaenians [31, 65], other fossorial reptiles [66–69], and also fossorial rodents [70], usually select microhabitats with sandy loose soils that are easier for burrowing. These patterns of soil selection might restrict the availability of areas suitable for amphisbaenians, and this limitation might initially be considered as a potential reason for the observed limited movements. However, in our study area, sandy soils preferred by this amphisbaenian [31] show a high uniform distribution in the study plots, being the most common of the soil types found. Therefore, the short distance movements and small areas covered cannot be explained by a low availability of optimal habitats (i.e., limited or patchy distribution of sandy soils easy to dig). Most, if not all, individuals recaptured could potentially had moved to much longer distances away from the initial capture points if the existence of “optimal” soils around was the only constraint.

Additionally, in an interspecific comparison, lizard home range size was shown to scale directly with energetic requirements [5]. Amphisbaenians and other fossorial reptiles have standard metabolic rates 34–67% below average for reptiles of their mass, which may be beneficial in their subterranean habitats [71], and these low energy requirements might also explain their low movement rates and small areas used [5, 7]. Finally, it is not unlikely that the high density of conspecifics in our study island sites in comparison with mainland sites (J. Martín personal observation) lso contributed to limit the movements of amphisbaenians, as it occurs in some lizards [57], and the situation might be different in mainland sites where density is lower. However, we observed a high overlap of individual locations, suggesting that maintaining “exclusive” territories was not a fundamental requisite, or that it was impossible to achieve given the high density of individuals.

Amphisbaenians were often recaptured under the same rocks where they were initially found, suggesting that they probably have high site fidelity and settle around “favorable” rocks or groups of rocks. Previous studies have shown that these amphisbaenians select rocks of an appropriate medium-size to thermorregulate under them, and because rocks maintain relatively higher humidity levels [30, 33, 53]. Also, the abundance and diversity of potential invertebrate prey found sheltering under rocks is higher than in open soil [35, 72]. Finally, many social interactions seem to occur under rocks [37, 40] and all of these benefits can be obtained while simultaneously being relatively protected by the rock from epigeal digging predators. This preference for using some rocks, and the fact that lifting rocks is an easy way for researchers to locate amphisbaenians, could have biased our surveys. Individuals might seem to have short movements just because they would be always found under the same preferred rocks, while if individuals would move long distances away from rocks, they could not be detected. However, the surveys using PIT telemetry allowed us to detect individuals also when they were underground in areas away from rocks. These surveys showed similar results to those based on recaptures under rocks alone, indicating that there was not a bias due to a likely preference for some rocks, and that the short distances observed reflect the actual space use of amphisbaenians.

Based on long-term data, it might be initially argued that amphisbaenians might simply move very slowly over very short distances in every single day, such that the final distance measured from capture to the first recapture after many days would be just the accumulative result of very short daily movements. However, a detailed analysis of data showed that this is unlikely because there was a lack of relationship between the number of days elapsed between successive observations and distance moved. Moreover, the short-term surveys indicated that, although some amphisbaenians did not seem to move in a meaningful way from 1 day to the next, even when environmental conditions were appropriate for activity, they were also able to move relatively long distances in a single day. In fact, many individuals detected were actively moving underground, and making relatively quick and long displacements in a few minutes while we tried to determine their location. This suggests that amphisbaenians might alternate quick bursts of movements, maybe from one preferred area to another while looking for food or mates, with relatively motionless stop-over periods of prey digestion or social interactions at favorable sites [37, 40].

Some intersexual and ontogenetic differences in movements were found in the study. The short-term surveys, made during the mating season, revealed that in comparison with females, males moved more days and over longer distances, although covered areas of similar size. This suggests that males have different reproductive requirements than females and probably have to move more frequently and for longer distances to locate potential mates, and perhaps to try to maintain territories relatively free of competitor males. However, these differences in movements did not seem to result in differences in areas covered. In contrast, in both Autarchoglossa (e.g., skinks, lacertids, anguimorphs, etc) and Iguania (e.g., iguanas, chamaeleons, agamids, etc) epigeal lizard species, males move more and also consistently have larger home ranges than females (see review in [5]). Future studies should examine the seasonal variation in movements and space use of male and female amphisbaenians using continuous focal observations, and the potential interactions with other nearby individuals.

In addition, we found some effects of body size/age on movements that might be explained by different ontogenetic related requirements and that also varied with sex. Specifically, smaller (younger) males moved more frequently and over longer distances than larger (older) ones, while females followed the opposite trend. With the current data, we can only speculate about the reasons for these size/age effects based on what is known for ontogenetic variation in social behavior of epigeal reptiles [5, 10, 15]. Thus, younger, but already adult, males might be more nomadic or floaters than older ones, which might be more settled in a given area and be more territorial. On the contrary, in females, simply different body-size-dependent food requirements might explain that larger individual females had to move more to find more prey. Further studies are clearly needed to test these hypotheses. The results of our study may also be relevant for conservation of amphisbaenians and fossorial reptiles in general. This is especially important because the lack of concern of conservationists for fossorial reptiles is notorious [17, 73], so many conservation threats may be occurring unnoticed [42, 43, 66, 74, 75]. The fact that these amphisbaenians move so little suggests that they have a limited mobility and dispersal capacity in comparison with other reptiles. Thus, damaging natural areas where these amphisbaenians live could directly endanger a population, because individuals could not be able to move and naturally recolonize undamaged or restored nearby areas [76]. Moreover, if there was a low dispersal ability it may result in low genetic diversity and high levels of inbreeding, which can in turn increase the risk of extinction of isolated populations [77, 78]. Therefore, data on movement and sapce use would be useful for managing populations and designing the size and location of nature reserves.

## Conclusions

We conclude that the low distance movements, small areas covered and the fact that almost one third of the days animals did not seem to move in a meaningful way indicate that *T. wiegmanni* amphisbaenians show high site fidelity. This spatial strategy could be explained by environmental constraints for moving underground, or the low energetic requirements of fossorial reptiles, but it also may favor the need to recognize familiar conspecifics and establish stable pairs and family groups as previous field observations suggested [37, 40]. Individual differences probably reflect differential reproductive and social requirements of males and females, and that younger individuals might show more floating behavior until they can settle in a territory. Our study is a rare example describing the spatial ecology of a fossorial species in an underground environment and may help to complete the understanding of the general factors that affect the space use and movement decisions in animals.

## Data Availability

The datasets supporting the conclusions of this article are available in the Figshare repository (10.6084/m9.figshare.14248067.v1).

## References

[1] Hansson LA, Akesson S (2014). Animal movement across scales.

[2] Tilman D, Kareiva P (2018). Spatial ecology: the role of space in population dynamics and interspecific interactions.

[3] Nathan R, Getzb WM, Revilla E, Holyoak M, Kadmon R, Saltz D, Smouse PE (2008). A movement ecology paradigm for unifying organismal movement research. Proc Natl Acad Sci U S A.

[4] Morris D (1987). Ecological scale and habitat use. Ecology.

[5] Perry G, Garland T (2002). Lizard home ranges revisited: effects of sex, body size, diet, habitat, and phylogeny. Ecology.

[6] Börger L, Dalziel BD, Fryxell JM (2008). Are there general mechanisms of animal home range behaviour? A review and prospects for future research. Ecol Lett.

[7] Tamburello N, Côté IM, Dulvy NK (2015). Energy and the scaling of animal space use. Am Nat.

[8] Boratyński Z (2020). Energetic constraints on mammalian home range size. Funct Ecol.

[9] Bonte D, Van Dyck H, Bullock JM (2012). Costs of dispersal. Biol Rev.

[10] Aragón P, López P, Martín J (2004). The ontogeny of spatio-temporal tactics and social relationships of adult male Iberian rock lizards, *Lacerta monticola*. Ethology.

[11] Bruinzeel LW, van de Pol M (2004). Site attachment of floaters predicts success in territory acquisition. Behav Ecol.

[12] Shaw AK (2020). Causes and consequences of individual variation in animal movement. Mov Ecol.

[13] Clobert J, Danchin E, Dhondt AA, Nichols JD (2001). Dispersal.

[14] Armsworth PR, Roughgarden JE (2005). The impact of directed versus random movement on population dynamics and biodiversity patterns. Am Nat.

[15] Rhodes JR, McAlpine CA, Lunney D, Possingham HP (2005). A spatially explicit habitat selection model incorporating home range behavior. Ecology.

[16] Measey GJ (2006). Surveying biodiversity of soil herpetofauna: towards a standard quantitative methodology. Eur J Soil Biol.

[17] Böhm M, Collen B, Baillie JEM, Bowles P, Chanson J, Cox N, Hammerson G, Hoffmann M, Livingstone SR, Ram M, Rhodin AGJ, Stuart SN, van Dijk PP, Young BE, Afuang LE, Aghasyan A, García A, Aguilar C, Ajtic R, Akarsu F, Alencar LRV, Allison A, Ananjeva N, Anderson S, Andrén C, Ariano-Sánchez D, Arredondo JC, Auliya M, Austin CC, Avci A, Baker PJ, Barreto-Lima AF, Barrio-Amorós CL, Basu D, Bates MF, Batistella A, Bauer A, Bennett D, Böhme W, Broadley D, Brown R, Burgess J, Captain A, Carreira S, Castañeda MR, Castro F, Catenazzi A, Cedeño-Vázquez JR, Chapple DG, Cheylan M, Cisneros-Heredia DF, Cogalniceanu D, Cogger H, Corti C, Costa GC, Couper PJ, Courtney T, Crnobrnja-Isailovic J, Crochet PA, Crother B, Cruz F, Daltry JC, Daniels RJR, Das I, de Silva A, Diesmos AC, Dirksen L, Doan TM, Dodd CK, Doody JS, Dorcas ME, Duarte de Barros Filho J, Egan VT, el Mouden EH, Embert D, Espinoza RE, Fallabrino A, Feng X, Feng ZJ, Fitzgerald L, Flores-Villela O, França FGR, Frost D, Gadsden H, Gamble T, Ganesh SR, Garcia MA, García-Pérez JE, Gatus J, Gaulke M, Geniez P, Georges A, Gerlach J, Goldberg S, Gonzalez JCT, Gower DJ, Grant T, Greenbaum E, Grieco C, Guo P, Hamilton AM, Hare K, Hedges SB, Heideman N, Hilton-Taylor C, Hitchmough R, Hollingsworth B, Hutchinson M, Ineich I, Iverson J, Jaksic FM, Jenkins R, Joger U, Jose R, Kaska Y, Kaya U, Keogh JS, Köhler G, Kuchling G, Kumlutaş Y, Kwet A, la Marca E, Lamar W, Lane A, Lardner B, Latta C, Latta G, Lau M, Lavin P, Lawson D, LeBreton M, Lehr E, Limpus D, Lipczynski N, Lobo AS, López-Luna MA, Luiselli L, Lukoschek V, Lundberg M, Lymberakis P, Macey R, Magnusson WE, Mahler DL, Malhotra A, Mariaux J, Maritz B, Marques OAV, Márquez R, Martins M, Masterson G, Mateo JA, Mathew R, Mathews N, Mayer G, McCranie JR, Measey GJ, Mendoza-Quijano F, Menegon M, Métrailler S, Milton DA, Montgomery C, Morato SAA, Mott T, Muñoz-Alonso A, Murphy J, Nguyen TQ, Nilson G, Nogueira C, Núñez H, Orlov N, Ota H, Ottenwalder J, Papenfuss T, Pasachnik S, Passos P, Pauwels OSG, Pérez-Buitrago N, Pérez-Mellado V, Pianka ER, Pleguezuelos J, Pollock C, Ponce-Campos P, Powell R, Pupin F, Quintero Díaz GE, Radder R, Ramer J, Rasmussen AR, Raxworthy C, Reynolds R, Richman N, Rico EL, Riservato E, Rivas G, da Rocha PLB, Rödel MO, Rodríguez Schettino L, Roosenburg WM, Ross JP, Sadek R, Sanders K, Santos-Barrera G, Schleich HH, Schmidt BR, Schmitz A, Sharifi M, Shea G, Shi HT, Shine R, Sindaco R, Slimani T, Somaweera R, Spawls S, Stafford P, Stuebing R, Sweet S, Sy E, Temple HJ, Tognelli MF, Tolley K, Tolson PJ, Tuniyev B, Tuniyev S, Üzüm N, van Buurt G, van Sluys M, Velasco A, Vences M, Veselý M, Vinke S, Vinke T, Vogel G, Vogrin M, Vogt RC, Wearn OR, Werner YL, Whiting MJ, Wiewandt T, Wilkinson J, Wilson B, Wren S, Zamin T, Zhou K, Zug G (2013). The conservation status of the world’s reptiles. Biol Conserv.

[18] Henderson RW, Powell R, Martín J, López P, Dodd CK (2016). Sampling techniques for arboreal and fossorial reptiles. Reptile ecology and conservation. A handbook of techniques.

[19] Gans C (1974). Biomechanics: an approach to vertebrate biology.

[20] Gans C (1978). The characteristics and affinities of the amphisbaenia. Trans Zool Soc Lond.

[21] Gans C (2005). Checklist and bibliography of the amphisbaenia of the world. Bull Am Mus Nat Hist.

[22] Navas CA, Antoniazzi MM, Carvalho JE, Chaui-Berlink JG, James RS, Jared C, Kohlsdorf T, Pai-Silva MD, Wilson RS (2004). Morphological and physiological specialization for digging in amphisbaenians, an ancient lineage of fossorial vertebrates. J Exp Biol.

[23] Baeckens S, García-Roa R, Martín J, Ortega J, Huyghe K, Van Damme R (2017). Fossorial and durophagous: implications of molluscivory for head size and bite capacity in a burrowing worm lizard. J Zool.

[24] Papenfuss TJ (1982). The ecology and systematics of the amphisbaenian genus *Bipes*. Occ Pap Calif Acad Sci.

[25] Colli GR, Zamboni DS (1999). Ecology of the worm lizard *Amphisbaena alba* in the cerrado of central Brazil. Copeia.

[26] Webb JK, Shine R, Branch WR, Harlow PS (2000). Life underground: food habits and reproductive biology of two amphisbaenian species from South Africa. J Herpetol.

[27] Andrade DV, Nascimento LB, Abe AS (2006). Habits hidden underground: a review on the reproduction of the Amphisbaenia with notes on four neotropical species. Amphib-Rept.

[28] Dial BE, Gatten RE, Kamel S (1987). Energetics of concertina locomotion in *Bipes biporus* (Reptilia: Amphisbaenia). Copeia.

[29] Bons J, Geniez P (1996). Amphibians and reptiles of Morocco.

[30] Civantos E, Martín J, López P (2003). Fossorial life constrains microhabitat selection of the amphisbaenian *Trogonophis wiegmanni*. Can J Zool.

[31] Martín J, López P, García LV (2013). Soil characteristics determine microhabitat selection of the fossorial amphisbaenian *Trogonophis wiegmanni*. J Zool.

[32] Gatten RE, McClung RM (1981). Thermal selection by an amphisbaenian, *Trogonophis wiegmanni*. J Therm Biol.

[33] López P, Civantos E, Martín J (2002). Body temperature regulation in the amphisbaenian *Trogonophis wiegmanni*. Can J Zool.

[34] Bons J, Saint Girons H (1963). Ecologie et cycle sexuel des amphisbeniens du Maroc. Bull Soc Sci Nat Phys Maroc.

[35] Martín J, Ortega J, López P, Pérez-Cembranos A, Pérez-Mellado V (2013). Fossorial life does not constrain diet selection in the amphisbaenian *Trogonophis wiegmanni*. J Zool.

[36] López P, Ortega J, Martín J (2014). Chemosensory prey detection by the amphisbaenian *Trogonophis wiegmanni*. J Herpetol.

[37] Martín J, Polo-Cavia N, Gonzalo A, López P, Civantos E (2011). Social aggregation behaviour in the north African amphisbaenian *Trogonophis wiegmanni*. Afr J Herpetol.

[38] Martín J, Polo-Cavia N, Gonzalo A, López P, Civantos E (2011). Structure of a population of the amphisbaenian *Trogonophis wiegmanni* in North Africa. Herpetologica.

[39] Martín J, Polo-Cavia N, Gonzalo A, López P, Civantos E (2012). Sexual dimorphism in the north African amphisbaenian *Trogonophis wiegmanni*. J Herpetol.

[40] Martín J, Raya-García E, Ortega J, López P (2020). How to maintain underground social relationships? Chemosensory sex, partner and self recognition in a fossorial amphisbaenian. PLoS One.

[41] Mateo JA, Joger J, Pleguezuelos J, Slimani T, Martínez-Solano I (2009). *Trogonophis wiegmanni*. The IUCN red list of threatened species 2009: e.T61589A12502172.

[42] Martín J, López P, Gutiérrez E, García LV (2015). Natural and anthropogenic alterations of the soil affect body condition of the fossorial amphisbaenian *Trogonophis wiegamnni* in North Africa. J Arid Environm.

[43] Martín J, Gutiérrez E, García LV (2017). Alteration effects of ornamental whitewashing of rocks on the soil properties and body condition of fossorial amphisbaenians that live under them. Herpetol Conserv Biol.

[44] Kuhnz LA (2000). Microhabitats and home range of the California legless lizard using biototelemtry. Ms theses.

[45] Connette GM, Semlitsch RD (2012). Successful use of a passive integrated transponder (PIT) system for below-ground detection of plethodontid salamanders. Wildlife Res.

[46] Ousterhout BH, Semlitsch RD (2014). Measuring terrestrial movement behavior using passive integrated transponder (PIT) tags: effects of tag size on detection, movement, survival, and growth. Behav Ecol Sociobiol.

[47] García LV, Marañón T, Ojeda F, Clemente L, Redondo R (2002). Seagull influence on soil properties, chenopod shrub distribution, and leaf nutrient status in semi-arid Mediterranean islands. Oikos.

[48] García LV (2005). Suelos de las Islas Chafarinas y sus relaciones ecológicas. Ecosistemas.

[49] Martín J, Polo-Cavia N, Gonzalo A, López P, Civantos E (2011). Distribución, abundancia y conservación de la culebrilla mora (*Trogonophis wiegmanni*) en las Islas Chafarinas. Bol Asoc Herpetol Esp.

[50] García-Roa R, Ortega J, López P, Civantos E, Martín J (2014). Revisión de la distribución y abundancia de la herpetofauna en las Islas Chafarinas: datos históricos vs. tendencias poblacionales. Bol Asoc Herpetol Esp.

[51] Gibbons JW, Andrews KM (2004). PIT tagging: simple technology at its best. Biosciences.

[52] Recio P, Rodríguez-Ruiz G, Ortega J, Martín J (2019). PIT-tags as a technique for marking fossorial reptiles: insights from a long-term field study of the amphisbaenian *Trogonophis wiegmanni*. Acta Herpetol.

[53] López P, Salvador A, Martín J (1998). Soil temperatures, rock selection and the thermal ecology of the amphisbaenian reptile *Blanus cinereus*. Can J Zool.

[54] Rose B (1982). Lizard home ranges: methodology and function. J Herpetol.

[55] Silva I, Crane M, Marshall BM, Strine CT (2020). Reptiles on the wrong track? Moving beyond traditional estimators with dynamic Brownian Bridge Movement Models. Mov Ecol.

[56] Diego-Rasilla FJ, Pérez-Mellado V (2003). Home range and habitat selection by *Podarcis hispanica* (Squamata, Lacertidae) in Western Spain. Folia Zool.

[57] Lecomte J, Clobert J, Massot M, Barbault R (1994). Spatial and behavioural consequences of a density manipulation in the common lizard. Ecoscience.

[58] Gent AH, Spellerberg IF (1993). Movement rates of the smooth snake *Coronella austriaca* (Colubridae): a radio-telemetric study. Herpetol J.

[59] Schmidt BR, Meier A, Sutherland C, Royle JA (2017). Spatial capture–recapture analysis of artificial cover board survey data reveals small scale spatial variation in slow-worm *Anguis fragilis* density. R Soc Open Sci.

[60] Barbour RW, Harvey MJ, Hardin JW (1969). Home range, movements and activity of the eastern worm snake, *Carphophis amoenus amoenus*. Ecology.

[61] Russell KR, Hanlin HG (1999). Aspects of the ecology of worm snakes *Carphophis amoenus* associated with small isolated wetlands in South Carolina. J Herpetol.

[62] Wu NC, Alton LA, Clemente CJ, Kearney MR, White CR (2015). Morphology and burrowing energetics of semi-fossorial skinks (*Liopholis* spp.). J Exp Biol.

[63] Seymour RS, Withers PC, Weathers WW (1998). Energetics of burrowing, running, and free-living in the Namib desert golden mole (*Eremitalpa namibensis*). J Zool.

[64] Luna F, Antinuchi CD (2006). Cost of foraging in the subterranean rodent *Ctenomys talarum*: effect of soil hardness. Can J Zool.

[65] Martín J, López P, Salvador A (1991). Microhabitat selection of the amphisbaenian *Blanus cinereus*. Copeia.

[66] How RA, Shine R (1999). Ecological traits and conservation biology of five fossorial ‘sand-swimming’ snake species (*Simoselaps*: Elapidae) in South-Western Australia. J Zool.

[67] Kuhnz LA, Burton RK, Slattery PN, Oakden JM (2005). Microhabitats and population densities of California legless lizards, with comments on effectiveness of various techniques for estimating numbers of fossorial reptiles. J Herpetol.

[68] Greenville AC, Dickman CR (2009). Factors affecting habitat selection in a specialist fossorial skink. Biol J Linn Soc.

[69] Martín J, García-Roa R, Ortega J, López P, Pérez-Cembranos A, León A, García LV, Pérez-Mellado V (2015). Occurrence and ecological aspects of the two-fingered skink *Chalcides mauritanicus* in the Chafarinas Islands in North Africa. Afr J Herpetol.

[70] Jackson CR, Lubbe NR, Robertson MP, Setsaas TH, van der Waals J, Bennett NC (2008). Soil properties and the distribution of the endangered Juliana’s golden mole. J Zool.

[71] Kamel S, Gatten RE (1983). Aerobic and anaerobic activity metabolism of limbless and fossorial reptiles. Phys Biochem Zool.

[72] López P, Martín J, Salvador A (1991). Diet selection by the amphisbaenian *Blanus cinereus*. Herpetologica.

[73] Decaens T, Jimenez JJ, Gioia C, Measey GJ, Lavelle P (2006). The values of soil animals for conservation biology. Eur J Soil Biol.

[74] Measey GJ, Armstrong AJ, Hanekom C (2009). Subterranean herpetofauna show a decline after 34 years in Ndumu game reserve, South Africa. Oryx.

[75] Tibbett M, Fraser TD, Duddigan S (2020). Identifying potential threats to soil biodiversity. PeerJ.

[76] Hromada SJ, Esque TC, Vandergast AG, Dutcher KE, Mitchell CI, Gray ME, Chang T, Dickson BG, Nussear KE (2020). Using movement to inform conservation corridor design for Mojave desert tortoise. Mov Ecol.

[77] Hedrick PW, Kalinowski ST (2000). Inbreeding depression and conservation biology. Annu Rev Ecol Syst.

[78] Frankham R (2005). Genetics and extinction. Biol Conserv.

